# Evidence of Knowledge Acquisition in a Cognitive Flexibility-Based Computer Learning Environment

**DOI:** 10.3885/meo.2008.Res00261

**Published:** 2008-12-03

**Authors:** Scott Heath, John Higgs, Daniel R. Ambruso

**Affiliations:** * Bonfils Blood Center, Denver, Colorado, USA; † Mid-State Technical College, Wisconsin, USA; ‡ University of Colorado School of Medicine, Aurora, Colorado, USA

**Keywords:** Clinical science education, learning theory, curriculum development, instructional simulation

## Abstract

**Background:**

A computer-based learning experience was developed using cognitive flexibility theory to overcome the pitfalls often encountered in existing medical education. An earlier study (not published) showed significant pretest-posttest increase in scores, as well as a significant positive correlation between choosing to complete the module individually or in pairs.

**Method:**

This experience was presented as part of a second-year course in medical school with randomized assignment for students to complete the program as pairs or individuals.

**Results:**

Sixty-six scores of 101 medical students (31 from students working as singles and 35 from 70 working in pairs) were analyzed. Out of 47 possible points, the mean pretest score was 15.1 (SD = 6.4, range 13.7-15.9). The mean posttest score was 22.9 (SD = 5.2, range 21.1-24.2). Posttest scores were statistically significantly higher than pretest scores (p<.001, Cohen's d = 1.17, average gain 7.8 points). Both pairs and singles showed pre-to-post test score gains, but the score gains of pairs and singles were not significantly different.

**Conclusion:**

This learning module served as an effective instructional intervention. However, the effect of collaboration, measured by score gains for pairs, was not significantly different from score gains of students completing the assignment individually.

Often the traditional teaching model produces learners unable to recall, use, or transfer knowledge and skills in novel situations. Whitehead^1^ calls this “inert knowledge” since it is information students in fact possess but that is not transferable to relevant situations because it is memorized devoid of context.^2^–^6^ Several theories and methods exist to establish learning environments that allow the knowledge gained to be useful in the relevant domain. Of special interest in this study was Cognitive Flexibility Theory^7^,^8^(CFT).

Feltovich, Spiro, and Coulson^9^ found that medical students had problems transferring knowledge previously learned in one context, such as medical school coursework, to new situations, such as clinical problem solving. Spiro and his colleagues attribute students’ deficiencies in learning outcomes (i.e., inert knowledge formation) at this advanced stage of learning to oversimplification (reductive bias) of complexity.^7^

A predominant share of the misconceptions (and networks of misconception) that we have identified reflect one or another kind of oversimplification of complex material …. Misconceptions of advanced material result from both interference from earlier, simplified treatments of that material and from a prevailing mode of approaching the learning process in general that fosters simplificational strategies and leaves learners without an appropriate cognitive repertoire for the processing of complexity.

Thus, ill-structuredness (the combination of breadth, complexity, and irregularity of a content domain) and the goals of advanced knowledge acquisition can, in combination, create a difficult problem for both teachers and students when they use simple strategies to present and learn complex subject matter. In response to this problem of reductive bias, Spiro and his colleagues proposed CFT as a means to create learning environments that provide the complexity and multidimensionality of content required for advanced knowledge acquisition in ill-structured domains.

## Background

The computer-based module “Handling Transfusion Hazards” was developed using these theoretical principles in a series of case-based instructional simulations for curriculum-specific content in Blood Banking and Transfusion Medicine. The principles of CFT (and their application to a specific content area of clinical medicine) as reflected in the development of this learning module for medical students included the following:^10^

- **Avoid oversimplifying instruction.** The complexity of multiple possibilities in terms of symptoms, causes, and data sources was represented.
- **Emphasize case-based instruction**. A variety of cases were chosen to avoid the reductive bias issues inherent in using a small number of prototypic cases.
- **Provide multiple representations of content**. In addition to history and laboratory tests, perspectives were available from several colleagues, basic information was found in a textbook, and a collection of “similar cases” was also provided.
- **Support context-dependent knowledge**. All events in these cases represent actual experiences of our advisors and so are well within the clinical context where they are likely to be encountered later by the learner.
- **Emphasize knowledge construction, not transmission**. Learners must construct complex schemata including procedural and factual knowledge in order to successfully complete each case.
- **Support complexity**. Multiple perspectives on and between cases allowed learners to experience some of the complexity of transfusion medicine.

We hypothesized that collaborative learning would further enhance the CFT-based education process. We evaluated this effect by organizing students to complete the module individually or in pairs and comparing test results for these groups. The collaborative learning process was seen as both consistent with these principles and a natural aspect of medical education and practice.

The program that was developed as a result was case-based, using a series of cases. Each case had a brief introduction, a report of the case history and physical examination, a range of typical laboratory tests from which to choose, assessment questions, and a range of management options. The practice cases also included a series of opinions and observations by colleagues (resident, blood bank director, etc.) and a bank of 24 similar cases that showed how such situations had been handled by other doctors. The test cases were presented in the same format but did not provide the extensive help functions available in the practice cases. In the test cases, students could be evaluated for their ability to assess and manage the clinical problem presented. Two transfusion medicine experts reviewed the test cases for content validity, comprehensiveness, and accuracy. Changes were made to the test cases based on the experts’ feedback. A simulated textbook was available during practice and test cases.

When the students encounter a list of cases to explore, and see the information resources available, they realize (or are reminded of) the complex, ill-structured nature of the transfusion medicine domain. This is the first example of how *“*Handling Transfusion Hazards” *avoids oversimplification* and *emphasizes case-based instruction*. The textbook, history with physical exam, perspectives from other people, and even the bank of similar cases, all provide *multiple representations of content*. As each case unfolds, the learners must use the non-compartmentalized information provided in clinical context to construct their own problem-solving approach, a process which *supports context-dependent knowledge, emphasizes knowledge construction,* and *supports complexity*.

**Research Questions** - The main question we sought an answer to was “Is completing the computer program ‘Handling Transfusion Hazards’ an effective way to learn topics in Transfusion Medicine?” No specific measure of sufficiency was established: any indication of increased performance on our test was of interest to us. A second question was “Does completing this program in collaboration with another student enhance its effect?” We also wanted to know how much time the students spent on the program and how they felt about the learning experience.

**Preliminary Study** - One year prior to the study described, we observed a class as they passed through the course. At this time, the computer program was piloted to determine if our anticipated measurements and interventions were practical and if medical students using a computer-based learning program would effectively collaborate and use this software. The pilot study participants were second-year medical students. The program used in the pilot study consisted of nine cases, two pretest cases, five practice cases, and two posttest cases. All students participated, and the material was effectively distributed to the students.

To determine if program users would effectively collaborate, five volunteer pairs were videotaped using the program. A coding scheme containing two main interaction types (on-task and off-task) and four verbal interaction categories (questions or statements for both interaction types) was developed as an observation checklist. The four interaction categories were further subdivided into two categories: (a) suggestions, opinions, directions, and (b) explanations, evaluations. The coding scheme also contained a provision for categorizing an interaction as either cognitive conflict (argumentative) or co-construction. The categories which the coders applied to each event in the interaction were On-task/Off-task, Question/Statement, Suggestion/Explanation, and Argumentative/Co-constructive.

Average intercoder agreement was .95. Although the sample was small, the data suggest that the medical students remained highly task-focused during the program phases observed. Furthermore, the interaction patterns demonstrate that the students concentrated their efforts in giving and receiving explanations, which are positive collaborative behaviors. Finally, the coders failed to document any cases of cognitive conflict, suggesting that these pairs created a co-constructive, collaborative environment. Since this portion of the preliminary study generated sufficient observational evidence that the students worked collaboratively, the investigators felt no need to further document that finding.

## Methods

**Scoring** – The module presents several clinical case-based scenarios^10^. The unscored cases are called practice cases and provide a full range of help resources: colleague perspectives, similar case synopses, and a reference textbook. In the test cases used for determining a score, the help is limited to the textbook (used if necessary), and each decision point (laboratory test, assessment fact, or management decision) results in a numeric value being added to (or subtracted from) the total score.

- Lab tests: Experienced practitioners assigned the scores as 2 (essential), 1 (possibly appropriate), 0 (irrelevant but harmless) or -1 (plainly mistaken, not relevant and inconvenient, expensive, or risky).
- Assessment: Each action selected for assessing a case received a score of -l or +1 depending on the validity and appropriateness of the selection.
- Management: Each management action received a score of −l or +1 depending on the appropriateness of the selection for the case.

The three test cases contained a total of 172 lab tests options, 19 assessment issues, and 16 management choices. Appropriate responses could earn 30 lab test points, 9 assessment points, and 8 management points for a total perfect score of 47 points. No score was determined as “adequate” and the scoring was not adjusted to reflect traditional percentage to letter-grade correspondence.

**Participants** - One hundred thirty-two second-year medical students, 70 men and 62 women, participated in this study. The subjects completed “Handling Transfusion Hazards” during the three-week Hematology segment of the Pathophysiology of Disease course. All subjects completed the program, but 31 students were excluded from the final analysis because of missing or inaccurate data. Thus, 101 students were included in final statistical analysis (31 singles and 35 pairs; 66 score reports).

**Design** – The experimental design employed the pretest-posttest control group design espoused by Campbell & Stanley,^11^ graphically depicted below (R = randomization, O = observation, X = treatment):

R O X_1_ O

R O X_2_ O

where X_1_ represents the unpaired condition and X_2_ represents the paired condition. Each subgroup can serve as control for the other and the pre-treatment observation provides a control for the entire population as described in the first paragraph of the discussion. The study's design established the submitted score as the unit of analysis rather than the individual student's score, so students assigned to the collaborative condition tested together rather than separately. Pairs achieved a single score that was analyzed along with scores earned by students completing the program as individuals. This allowed the pretest results to be easily compared to the posttest results, as all subgroups (scores from pairs and scores from singles) were handled identically.

The scores on the pretest and posttest were based on the lab tests ordered and how the three test cases were assessed and managed. These cases were conceptually similar to the cases presented during the learning phase of the program.

**Random Assignment** – For the main study, students were randomly assigned to work individually or in pairs so that roughly 1/3 would work individually and 2/3 of the class would form pairs. This would yield about as many scores from individual students as from pairs.

**Lecture Hall Intervention** – During a 30-minute lecture on the first day of the hematology section, the students were introduced to the computer module. In this lecture, the transfusion medicine curriculum manager discussed the program content and learning objectives, the logistics of obtaining a computer disk, the requirement to complete the program, and grading criteria. The students were also told that they must complete the program as assigned (i.e., if assigned to work individually, they must not consult with any other student).

**Program Structure** - As a result of the non-equivalent nature of the four test cases used in the pilot study, one test case was re-designed to fit into the practice cases section. The remaining three test cases (referred to as A, B, and C) were used as both a pretest and a posttest, thus assuring equivalence. The portion of score differences attributable to testing effects was considered to be insignificant. The case structure now consisted of the three test cases as a pretest, five practice cases, and then the same three test cases as a posttest.

**Data Collection and Statistical Analysis**-The data disks recorded pretest and posttest results (selections and scores), as well as other data such as time information, student explanations and summaries, and program critiques. These data were extracted from the disks, checked for accuracy, and hand entered into the Statistical Package for the Social Sciences (SPSS 7.5 for Windows) data editor. This database served as the master file for the statistical analyses. This study examined the effects of collaboration on knowledge acquired by advanced learners in a cognitive flexibility theory-based computer microworld. The independent variable was method of instruction (single or pairs). The dependent variable was the score achieved on a posttest consisting of three transfusion medicine cases. Analysis of covariance (ANCOVA) was used to test the experimental hypotheses: scores will improve from pretest to posttest and scores from pairs will show greater improvement. Further evaluation using a spreadsheet with appropriate formulas for Cohen's d^12^ provided effect size measures to evaluate practical significance.

**Student Comments** - Subjects were given the opportunity to provide constructive feedback after finishing the program. These comments were independently coded by two raters into categories such as Time or Instructional Method. Where disagreement occurred, the raters discussed and revised their codes to achieve consensus.

## Results

**Score information, learning time, and program effectiveness** - A total of 66 scores were analyzed. These scores came from 101 medical students, 31 from students working as individuals and 35 from the 70 students working in pairs. Out of 47 possible points, the overall mean pretest score was 15.1 and the overall mean posttest score 22.9. The total posttest scores were statistically significantly higher than pretest scores (p<.0001 by the ANCOVA), showing an average gain of 7.8 points. Both pairs and singles showed statistically significant (p<.0001) and very large (d = 1.25 singles, d = 1.44 for pairs) pre-post test score gains (see Table 1), but the score gains of pairs and singles were not statistically significantly different from each other (p=.943 for the pairs/singles ANCOVA). The time for completing the 5 learning cases was 72.2 minutes + / − 30.8 minutes (mean + / − SD). Completion of the pretest, learning cases, and posttests ranged from 63.0 to 88.8. No difference was noted between groups of scores from singles or pairs. Table 1 presents scores and descriptive statistics for this investigation.

**Table 1:** Pre/Post test scores and time spent in practice phase (mean + / − SD)*

Submitted by	Pretest score	Post-test score	Practice case minutes
Pair	15.6 + / − 5.2	23.1 + / − 5.2	70.5 +/ − 29.6
Single	14.5 + / − 7.7	22.7 + / − 5.2	74.1 + / − 32.5
Overall	15.1 + / − 6.4	22.9 + / − 5.2	72.2 + / − 30.8

*Maximum score = 47, n = 66

The results of interest from statistical analyses were the values of F and p for the singles/pairs ANCOVA, which indicated that interaction of the scores from the single versus paired students was not statistically or practically significant (F=.01, p=.943). By calculating effect size from the F statistic and the sample size^12^, a value for Cohen's d of 0.04 was obtained, representing an effect of < 1%. However, the interaction between the covariate (pretest total score) and the dependent value (posttest total score) yielded F = 21.72 and p <.0001, which renders a d of 1.17, a very large effect.

**Analysis of Comments** - Table 2 presents the students’ feedback. The subjects’ responses were negative regarding the time required of them and the program, as as an instructional method. The subjects’ responses were positive regarding the opportunity and benefits of collaborative learning.

**Table 2:** Student Comments

Category of Feedback		Number of Responses
Time	Too much time	15
	Computers too slow	8
	Not enough time	2
Instructional Method	Do not like computer-based instruction	8
	Prefer lecture	11
	Did not learn from program	10
	Posttest cases differ from practice cases	15
	Program is a good educational tool	8
Program Specific Feedback	Lack of immediate feedback on test cases	14
	Inconvenient access to information sources	4
	Inability to change answers	9
Collaboration	Preferred	13
	Not preferred	1

## Discussion

Cognitive flexibility theory (CFT) has been advanced as a means to overcome the problem of inert knowledge formation by advanced learners in complex and ill-structured knowledge domains^13^. The research to date supports the assertion that CFT-based environments promote greater knowledge acquisition and transfer than linear treatments of the same subject content.^14^–^16^ Although compromises in research design (such as the self-controlling subject population) were required by the real-life setting, the results presented here suggest that “Handling Transfusion Hazards” is another CFT-based experience that promotes learning.

Effect size measures reported in the previous section indicate that both statistically and practically significant amounts of learning took place. The statistically insignificant and negligible effect size estimates regarding collaboration, despite the encouraging observational data from the preliminary study, indicates that confounding factors may have suppressed the hypothesized effect. Ensuring a strictly random assignment to the paired condition, for instance, may have interfered with an essential component of forming successful collaborative groups such as unrestricted choice of partners. In any event, score gains for pairs did not appear to differ significantly from those of singles, and students engaged in this program as singles or pairs performed equally well in test cases.

**Recommendations for Future Research** - This study raises several areas for further research. A qualitative approach might yield more useful insight into the dynamics of knowledge acquisition with such case-based instructional simulations. A larger question must be answered regarding the integration of cognitive flexibility theory or any other active, student-centered learning environment into the medical education arena. The students studied were accustomed to memorizing and reproducing information and may not have been prepared to deeply analyze and synthesize the information. The learning program might be better accepted in the third year where clinical evaluation, including interpretation of laboratory tests, and managing cases becomes the predominant approach to learning. This study points to the need to address the question of what conditions are necessary to successfully implement an active, student-centered learning environment.
